# Outlines of Structural and Physiological Botany

**Published:** 1847-07

**Authors:** 


### Art. XV.

*Outlines of Structural and Physiological Botany.* By Arthur Henfrey, F.L.S., &c., Lecturer on Botany at St. George's and the Middlesex Hospitals.—*London,* 1847. Fcp. 8vo, pp. 298. With Eighteen Plates.

The rapid advance of botanical science, and especially of that portion of it relating to the structure and growth of the primary tissues, renders the appearance of a new elementary treatise, from the pen of a gentleman who is well known to have devoted great attention to the study, extremely welcome; and a careful examination of the result of his labours has only increased our satisfaction, in almost every particular. The following quotation from his preface will show that he is fully alive to the importance of recent discoveries; and that he is by no means one of those writers of manuals, who take their cue from the antiquated treatises of their predecessors.

"In the natural sciences, especially, the accession of new information, whether from original investigation or from the persevering application of repeated examination and comparison to received views, is continually modifying the very fundamental doctrines on which our superstructure of knowledge temporarily rests; and thus, at certain stages, arises the necessity for the revision of the whole, the rejection of superseded and superfluous disquisitions, and for the assumption of new points of view, from which to regard much of that with which we have been previously acquainted. From such and analogous causes originates the production of new books of the nature of the present; and in them is to be found the justification of those who devote themselves to such tasks."

The first chapter contains a condensed account of the chemical constituents of plants; and in the second, the author proceeds to treat of their elementary structure, commencing with "the cell as an individual," and then proceeding to "cells in connexion, or tissues." In this manner, starting with the simple ideas derived from the consideration of the structure and actions of the single cell, he proceeds synthetically to build up (as it were) the entire plant, with its several organs and its distinct series of operations. Mr. Henfrey has bestowed considerable attention on the process of multiplication of cells, as it takes place in the plant, and has presented to the British Association more than one valuable communication on the subject. We shall endeavour to convey an idea of his doctrine, although this is difficult without the aid of figures. The wall of all young cells is lined by a very delicate membrane; which is termed by Mohl (who first pointedly directed attention to it as a general constituent of the cell)

the "primordial utricle." It is easily brought into view by tincture of iodine, which causes it to become brown and to contract, so as to detach it from the outer wall of the cell. We had ourselves observed this inner membrane long before it was described by Mohl; but had supposed it confined to the cells of the epidermis, in which alone we had met with it. Now, according to Mr. Henfrey, the division of a parent-cell into two (which is the usual method of multiplication, except when distinct germs are formed within it and set free by its rupture) is accomplished after this manner. The primordial utricle folds inwards, as if it were constricted by a cord passed around it; and the fold gradually grows inwards towards the centre, separating the cavity of the cell into two parts, which at last become perfectly closed and distinct sacs. Each lamella of the primordial utricle deposits a layer of matter on its exterior, constituting the permanent cell-wall; and thus a complete partition, continuous with the external cell-wall, is formed between the two newly-generated cells. This we believe to be the exact representation of the mode in which the multiplication of the cells of cartilage takes place; but whether it is the universal method of cell-generation in the animal and vegetable tissues, it would be premature yet to assert. The nucleus does not seem to perform any important part in these changes; and Mr. Henfrey believes with Mohl that it is intimately connected with the chemico-organic changes of the cells-contents, and especially in the elaboration of the mucilaginous granular matter, which has been well named by Mohl the "protoplasma" of the cell. Our readers will have noticed the recent tendency, among animal physiologists, to regard the nucleus as rather the essential agent in the chemico-organic changes, than as having any necessary connexion with the reproduction of the cell; and it is interesting to see how these two conclusions, founded upon such remote data, tend to confirm each other.

In the third chapter we find some "General Considerations on the Physiology of the Elementary Structures;" in which are embodied many facts of very great interest. From these we may quote the following concise statement of a phenomenon which has lately attracted much attention, and which will probably lead to an entirely new view of the character of the greater part of the so-called polygastric animalcules.

"The spores of some of the lower Algæ present some remarkable phenomena of motion. They consist of cellules furnished with appendages resembling cilia, which enable them to swim about in water like animals. Thuret and Kützing have found in these a red spot resembling the so-called eye of Ehrenberg's green monads. The fact of the development of these spores from undoubted plants like the Confervæ, goes far to prove that the Diatomaceæ, &c., and even Volvox and its allies, are plants. In this case, the existence of spontaneous motion in plants would be a well-marked character of vegetable life; but we are still in the dark as to the causes and nature of such phenomena."

The fact of the presence of cilia on cells of undoubted vegetable character, and of the performance of regularly continued movements by these bodies,—the absence of any definite character by which the movements of the so-called polygastric animalcules may be distinguished,—the strong resemblance between the general characters and mode of multiplication of beings of that class, and those of the undoubted plants on the one hand, and the animal ovum in its earliest stages on the other,—seem, when taken collectively, to warrant the doubt whether any adequate proof exists of the presence of *consciousness* in the Polygastrica, and whether we ought not either to exclude them from the animal kingdom, or to extend our notion of an animal to beings unpossessed of sensation; and if the latter alternative be adopted, we cannot see how any definition shall be framed that shall separate the animal from the vegetable. Perhaps, after all, it would be better not to attempt to carry such a separation through the lowest forms of organic existence, which present such a striking resemblance to the early embryonic conditions of the higher; and to classify those beings, in which no definite nervous system can be discovered, but which exhibit determinate movements, rather in conformity with their general organization and with their mode of reproduction, than by any arbitrary assumption regarding their possession of consciousness.

The remaining portion of the work is occupied with the consideration of the structure and physiology of plants considered as individuals; and we cannot too highly praise the manner of treating the various departments of these subjects, which is as remarkable for its clearness as for its truly philosophical character. The Cryptogamia are assigned their true rank in the scale of physiological importance,—for the first time, we believe, in any English botanical treatise claiming for itself a scientific value; and the author has brought together a large amount of new matter regarding their structure and actions, from the observations of eminent continental botanists, which will be new to the greater part of our readers. The only fault we have to find is with the illustrations, which, though very numerous and well-selected, are deficient in clearness, and by no means keep up the credit of Mr. Van Voorst's beautiful series of publications. They have the advantage, however, of being drawn by Mr. Henfrey himself; and their scientific correctness (a point about which mere artists often care but very little) is thus guaranteed.

---

